# Antibodies to Transglutaminase-6 Identify Ataxia Responsive to Gluten-Free Diet

**DOI:** 10.3390/nu18162645

**Published:** 2026-08-13

**Authors:** Marios Hadjivassiliou, Richard A. Grünewald, Iain D. Croall, Nigel Hoggard, Graeme Wild, Panagiotis Zis, Priya Shanmugarajah, David S. Sanders

**Affiliations:** 1 Academic Department of Neurosciences, Sheffield Teaching Hospitals NHS Trust, Sheffield S10 2JF, UK; grunewaldrichard@gmail.com (R.A.G.); priya.shanmugarajah1@nhs.net (P.S.); 2 Department of Infection and Immunity, University of Sheffield, Sheffield S10 2JF, UK; i.croall@sheffield.ac.uk (I.D.C.); n.hoggard@sheffield.ac.uk (N.H.); 3 Department of Immunology, Sheffield Teaching Hospitals NHS Trust, Sheffield S10 2JF, UK; graeme.wild1@nhs.net; 4 Medical School, University of Cyprus, Nicosia 2029, Cyprus; zis.panagiotis@ucy.ac.cy; 5 Department of Gastroenterology, Sheffield Teaching Hospitals NHS Trust, Sheffield S10 2JF, UK; david.sanders1@nhs.net

**Keywords:** gluten ataxia, cerebellar ataxia, TG6 antibodies, coeliac disease, gluten-free diet

## Abstract

**Background:** Gluten ataxia (GA) is the most common neurological manifestation of gluten sensitivity. GA is identified by serological evidence of gluten sensitivity when other defined causes for ataxia have been excluded. Whilst there are several serological markers for gluten sensitivity, antibodies against transglutaminase enzyme 6 (anti-TG6) are of particular interest as it is the autoantigen in GA. Our objective was to assess the value of a commercial anti-TG6 ELISA assay in routine clinical practice for the investigation and management of patients with progressive ataxia. **Methods:** Patients with progressive ataxia underwent standardized clinical and detailed investigative workup. Anti-TG6 was measured using a commercial ELISA assay. Patients with ataxia who were seropositive for any gluten sensitivity-related antibody were offered dietary advice for a gluten-free diet. All patients underwent baseline and follow-up MR spectroscopy scans of the cerebellum. **Results:** 155/161 (96%) patients with GA diagnosed on the basis of circulating antigliadin antibodies (the original definition of gluten ataxia) were also positive for anti-TG6. In total, 329/425 (77%) patients with idiopathic sporadic ataxia, all other causes of ataxia excluded, were positive for anti-TG6 alone. 115/296 (39%) patients with ataxia due to genetic causes were positive for anti-TG6, as were 17/59 (29%) patients with a clinically established cerebellar variant of multi-system atrophy. Amongst patients with other neurological conditions (non-ataxia), anti-TG6 was found in 13%, compared with 50% of patients with coeliac disease presenting to gastroenterologists. Overall, 146 patients with ataxia and anti-TG6 alone responded to a gluten-free diet, with a reduction/elimination of TG6 antibodies and improved MR spectroscopy. Patients with an identified genetic ataxia and anti-TG6 did not show a significant response to a gluten-free diet. **Conclusions:** Anti-TG6 appears to be a more sensitive diagnostic marker for GA when compared to antigliadin antibodies. Patients with ataxia and isolated anti-TG6 benefit from a strict gluten-free diet.

## 1. Introduction

Cerebellar ataxia is the most common neurological manifestation of gluten sensitivity with and without enteropathy-coeliac disease (CD) and responds to a strict gluten-free diet (GFD) [1]. The diagnosis of gluten ataxia (GA) was originally based on the presence of cerebellar ataxia in the context of seropositivity for antigliadin antibodies (AGA; IgG and/or IgA), irrespective of the presence of gluten-sensitive enteropathy (CD), and after exclusion of other causes of progressive ataxia [1]. Where enteropathy is present, seropositivity for transglutaminase 2 (also known as tissue transglutaminase-TTG) and endomysial antibodies (an immunofluorescence assay that also detects TTG) would be expected. 

The term gluten sensitivity (GS) as applied to the neurological cohort of patients encompasses not just those patients with CD who present with or develop neurological dysfunction but also patients with neurological dysfunction who are seropositive for AGA without enteropathy. Both groups (with and without enteropathy) have been shown to be neurologically clinically indistinguishable and to respond equally well to strict GFD.

An immunological response directed against different types of transglutaminases holds a clue to the diversity of clinical manifestations (gastrointestinal, dermatological, neurological). Transglutaminase type 2 was identified as the autoantigen in CD, whilst TG3, an epidermal transglutaminase, was shown to be the autoantigen in dermatitis herpetiformis [2,3].

We have previously shown that TG6, a brain-expressed transglutaminase, is an autoantigen in GA, that anti-TG6 antibodies (both IgG and IgA) are prevalent in GA and that accumulation of IgA deposits in human cerebellum of GA brain co-localizes with TG6 [1].

The clinical utility of TG6 antibodies in routine clinical practice in the context of the diagnosis of GA has not been fully explored. The development of a commercial assay for TG6 antibody testing (Zedira GmbH, Darmstadt, Germany) has facilitated its use. As a specialist ataxia centre which assesses patients with ataxia from all over the UK, the Sheffield Teaching Hospitals NHS Trust introduced the commercial TG6 assay for clinical use alongside other gluten sensitivity-related serological tests already readily available in our immunology laboratory (AGA, TTG and endomysium antibodies (EMA)). We present here our experience with the clinical utility of TG6 antibody testing.

## 2. Materials and Methods

This retrospective data analysis was given ethical approval via the East Midlands REC (IRAS 327729). Informed consent was waived because the study was retrospective and reported on outcomes based on standard clinical care.

### 2.1. Patient Selection and Investigations

The Sheffield Ataxia Centre is one of 3 UK-accredited National Ataxia Centres, and a member of the NHS Rare Diseases Clinical Network, receiving referrals from all over the UK. All patients with progressive cerebellar ataxia attending the Sheffield Ataxia Centre (currently >3500 patients) undergo detailed clinical examination with particular emphasis on cerebellar functioning (ocular signs, speech, limb and gait ataxia) and undergo an extensive battery of tests looking for causes of progressive ataxia including both genetic (whole genome sequencing, testing for Cerebellar Ataxia, Neuropathy and Vestibular Areflexia syndrome (CANVAS) and spinocerebellar ataxia type 27B-SCA27B) and acquired causes, as part of a standard clinical workup. The serological screening for GS includes anti-EMA (Euroimmun, Lubeck, Germany, Monkey Intestine slides using immunofluorescence), anti-TTG (Phadia, Uppsala, Sweden, positive if >7 U/mL), AGA (IgG and IgA, Organtec Diagnostika, Mainz, Germany, positive if >12 U/mL) and, as of June 2019, anti-TG6 antibodies (Zedira GmbH, Germany, IgG and IgA, positive IgG > 5.1, positive IgA > 4 U/mL). Those seropositive for one or more of these antibodies, in whom other causes of ataxia have been excluded, are offered dietary advice and encouraged to adopt GFD by dietitians experienced in the field of gluten sensitivity and CD.

Prior to the COVID-19 pandemic, all patients seropositive for one or more of these antibodies were referred for duodenal biopsy. Following the limitations in accessibility to endoscopy services during the COVID-19 pandemic, and the more recent guidelines in relation to the diagnosis of CD based on serological screening alone, only a small number of patients have undergone gastroscopy and duodenal biopsy and none had any evidence of coeliac disease [4,5,6,7]. The beneficial effect of GFD in patients with GA diagnosed on the basis of positive AGA with or without enteropathy has been reported previously.

### 2.2. Imaging

All patients routinely undergo MR spectroscopy of the cerebellar vermis and right hemisphere before (baseline) and after GFD to provide information on cerebellar *N*-acetyl-aspartate/Creatine (NAA/Cr) area ratio in each region. NAA reflects healthy metabolic activity whilst creatine is a stable metabolite. Reduction in NAA reflects the presence of cerebellar dysfunction. There is good correlation between NAA/Cr and the Scale for the Assessment and Rating of Ataxia (SARA score). Cerebellar vermis spectroscopy is much more reliable and reproducible than hemisphere spectroscopy because the voxel of interest can easily be positioned reproducibly within well-defined anatomical boundaries. In cerebellar hemisphere spectroscopy, the surface area available for voxel positioning is very large and such positioning can vary from scan to scan, compromising reproducibility. In the case of GA, which is an immune-mediated ataxia with preferential involvement of the vermis, vermian spectroscopy is ideal for monitoring and evaluating treatment responses.

### 2.3. Statistical Analysis

Mean, standard deviation, standard error and box-and-whisker statistics were calculated using an Excel spreadsheet. Correlation analysis was undertaken using an online tool for parametric and non-parametric analysis https://www.statskingdom.com/correlation-calculator.html (accessed 16 July 2026).

## 3. Results

### 3.1. Correlation Between AGA and TG6 Antibodies in GA

One hundred and fifty-five out of 161 patients (96%) with GA diagnosed on the basis of the presence of circulating AGA in the absence of an alternative cause for their ataxia (the original definition of GA) were seropositive for TG6 IgA and/or IgG antibodies at baseline testing. The majority (81%) were seropositive for IgA TG6 antibodies. The mean age at onset of the ataxia in this group was 53 (range 2–83 years). The mean duration of the ataxia was 12 years (range 5–59). Correlation analysis using a non-parametric test (Spearman’s rank) between TG6 IgA and antigliadin IgA antibody titre (range for TG6 was 4.1 to 100 U/mL, and for antigliadin antibodies 12 to 213 U/mL) showed a highly significant correlation (correlation coefficient 0.22 with a *p* = 0.009). For IgG antibodies, the correlation coefficient was 0.17 and of marginal significance at *p* = 0.06.

### 3.2. Prevalence of TG6 Antibodies in Other Ataxia Groups

Amongst 356 patients diagnosed with GA before the TG6 assay became available, and already on GFD, regularly attending our centre, 236 (66%) were seropositive for TG6 antibodies (IgA and/or IgG) when first tested for TG6 antibodies, 54% for IgA TG6 (no baseline samples for TG6 testing were available before the introduction of GFD). From the same group, 133 (37%) were seropositive for AGA (by definition, 100% were seropositive for AGA at baseline testing). This group also had the same clinical characteristics as the first group mentioned before.

Amongst 425 patients with idiopathic sporadic ataxia (ISA), in whom all other causes of ataxia had been excluded (including extensive genetic testing using whole-genome sequencing (WGS) as well as testing for SCA27B and CANVAS, 329 (77%) were seropositive for TG6 antibodies alone (IgA and/or IgG), with 64% being positive for IgA TG6 with or without IgG. These patients had no other serological evidence of gluten sensitivity (seronegative to AGA IgG and IgA, TGG, EMA). We found no correlation between TG6 titre and duration of the ataxia. This finding suggests that TG6 antibodies may be a more sensitive marker in defining and diagnosing GA.

Two hundred and ninety-six patients with ataxia subsequently proved to have an identified genetic cause. This was a heterogeneous group of patients with 40 different genetic aetiologies for their ataxia. 115 (39%) were seropositive for TG6 antibodies (33% IgA with or without IgG) and 23% were seropositive for AGA antibodies. Listed in order of prevalence in our population, 7 of 32 patients with SCA27B, 14/32 with Friedreich’s ataxia, 12 of 29 with Episodic Ataxia type 2, 12/28 with SPG7, 12 of 24 with CANVAS due to RFC1 mutations, 7 of 22 with SCA6 and 7 of 14 with SCA48 were seropositive for TG6 antibodies. All the patients from this group who were seropositive for anti-AGA were also seropositive for TG6 antibodies.

Amongst 59 patients with clinically established cerebellar variant of multi-system atrophy (MSA-C), a degenerative form of progressive ataxia, 17 (29%) were seropositive for TG6 (19% IgA with or without IgG). The mean age at symptom onset for this group was 58 years, with a mean disease duration to death of 7 years.

Although there was no significant difference in the prevalence of TG6 antibodies amongst the different types of genetic ataxias, there was a significant difference in the prevalence of TG6 antibodies between genetic ataxias and MSA-C (*p* = 0.042 by Chi-square), with MSA-C patients having a lower prevalence of TG6 antibodies. The same was true when comparing genetic ataxias and the other neurology group.

We investigated any possible correlation between the prevalence of TG6 antibodies and the duration of the genetic ataxia. We focused on a genetically homogeneous group of 23 patients with SCA6 because this is one of the commonest genetic ataxias in the UK, is late onset, usually pure ataxia with a reasonably well-defined age at symptom onset (unlike ataxias with onset in childhood), as well as having a long duration of disease follow-up at our ataxia centre. We found a significant difference in the prevalence of TG6 antibody positivity in those with long duration (higher prevalence) vs those with short duration (75% negative at <10 years duration of ataxia, 10% negative > 10 years duration of ataxia, *p* = 0.002 by Chi Square test). We found a weak correlation trend between IgA TG6 antibody titre and duration of the ataxia using Pearson’s correlation test (*p* = 0.08) (not significant with Spearman’s rank). For IgG TG6 antibodies, there was also a trend (*p* = 0.09, Pearson’s correlation test) between duration of the ataxia and titre of TG6 antibodies, and this was significant when using Spearman’s rank (*p* = 0.017, correlation coefficient 0.8).

### 3.3. Prevalence of TG6 in Non-Ataxia Groups

Amongst a control group of patients with neurological conditions other than ataxia undergoing immunological screening that included TG6 testing, 20/151 (13%) were seropositive for TG6 (11% IgA with or without IgG) antibodies and 5% were seropositive for AGA. This was a heterogeneous group of patients with vasculitis, neurosarcoidosis, connective tissue diseases, demyelinating neuropathies, immune encephalitides and other, mainly inflammatory, conditions. The figure is significantly less than the prevalence of TG6 seropositivity in those with a proven genetic cause for their ataxia (39%) and those with GA (96%) (*p* < 0.0001 by Chi-square test).

Seropositivity for TG6 antibodies was found in 25/50 (50%) patients with newly diagnosed CD (38% IgA with or without IgG) presenting to the gastroenterologists.

### 3.4. Effect of GFD in Patients with Ataxia and Isolated Seropositivity for TG6 Antibodies

The mean age at onset of the ataxia in this group of 146 patients (these were amongst the 425 patients with idiopathic sporadic ataxia screened and positive for TG6 antibodies, see above) was 54 (range 6–80 years) and the mean duration of their ataxia was 18 (range 4–50 years). A clinical and spectroscopic improvement following adoption of GFD in patients with GA (defined by serological AGA seropositivity and no other cause for ataxia, with or without enteropathy) has already been reported.

These 146 patients with positive TG6 antibodies but negative EMA, TGG and AGA underwent baseline and follow-up scans (mean: 22.2 months between each scan, range: 6–80 months) before and after the introduction of GFD. In 91 out of 146 patients (62%), the TG6 antibody titre became seronegative on adoption of GFD at the time of the second scan and in 42/146 (29%) it decreased, but was not seronegative. In 9 patients (6%) the titre increased and in 4 patients there was no record of a second sample being taken.

**Figure 1:**
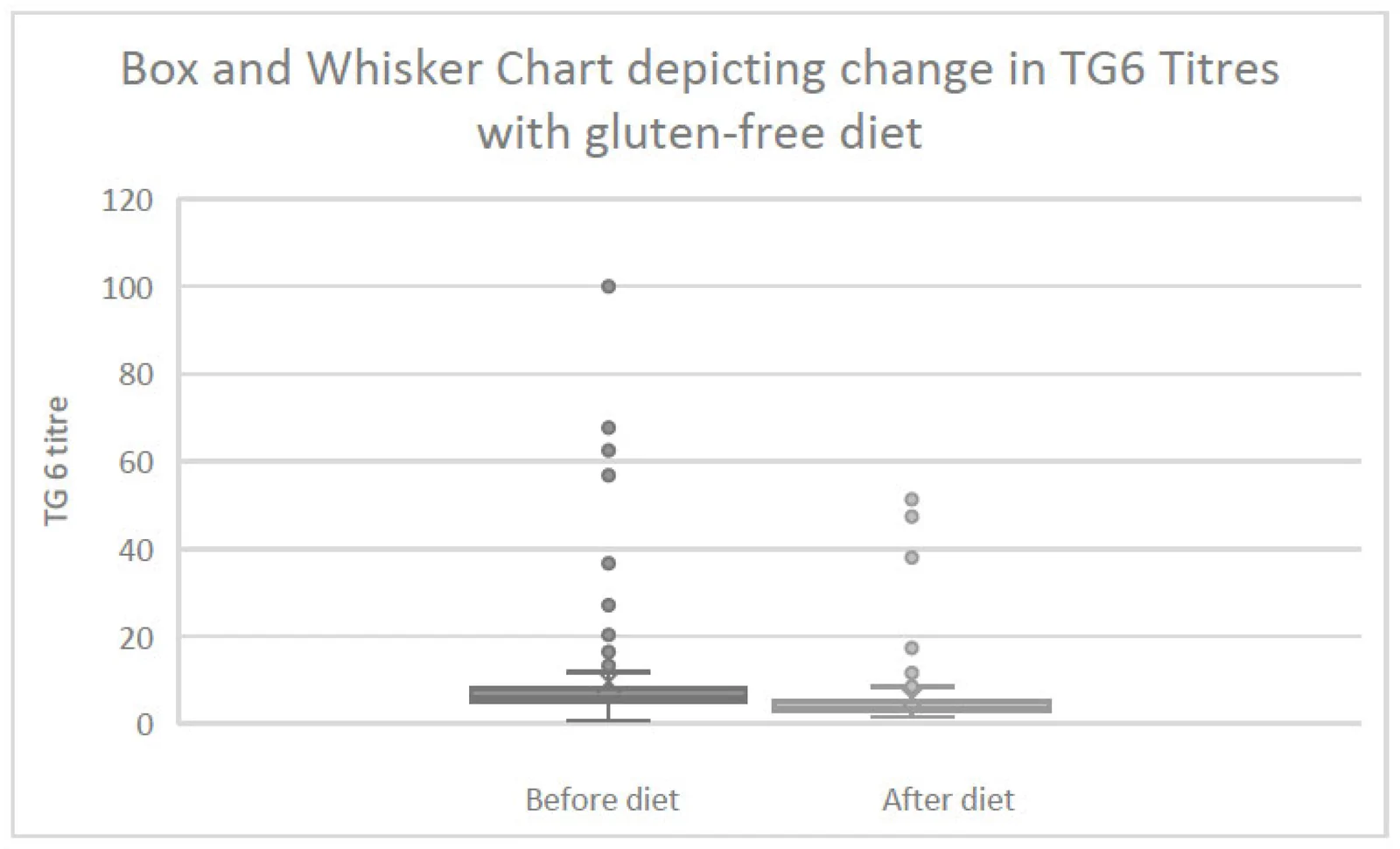
Box-and-whisker chart of change in the TG6 titre (IgA or IgG if IgG only was positive) in 146 patients with idiopathic sporadic ataxia and isolated seropositivity for TG6 antibodies. The TG6 antibody titre from baseline to the time of the second scan (mean 22.2 months between the 2 scans) fell significantly after introducing the GFD: In 91 out of 146 patients (62%), the TG6 antibody titre became seronegative and in 42/146 (29%) it decreased. In 9 patients (6%), the titre increased and in 4 patients there was no record of a second sample being taken (median titre before diet for the whole group: 5.9; range: 0.7–100; after diet: 3.6; range: 1.6 to 53; *p* < 0.00001 Wilcoxon signed rank test).

The TG6 antibody titre from baseline to the time of the second scan (after introduction of GFD) fell significantly (median titre before diet: 5.9; range: 0.7–100; after diet: 3.6; range: 1.6 to 53; *p* < 0.00001 Wilcoxon signed-rank test). The change in the TG6 titre in this cohort is illustrated in Figure 1.

**Figure 2:**
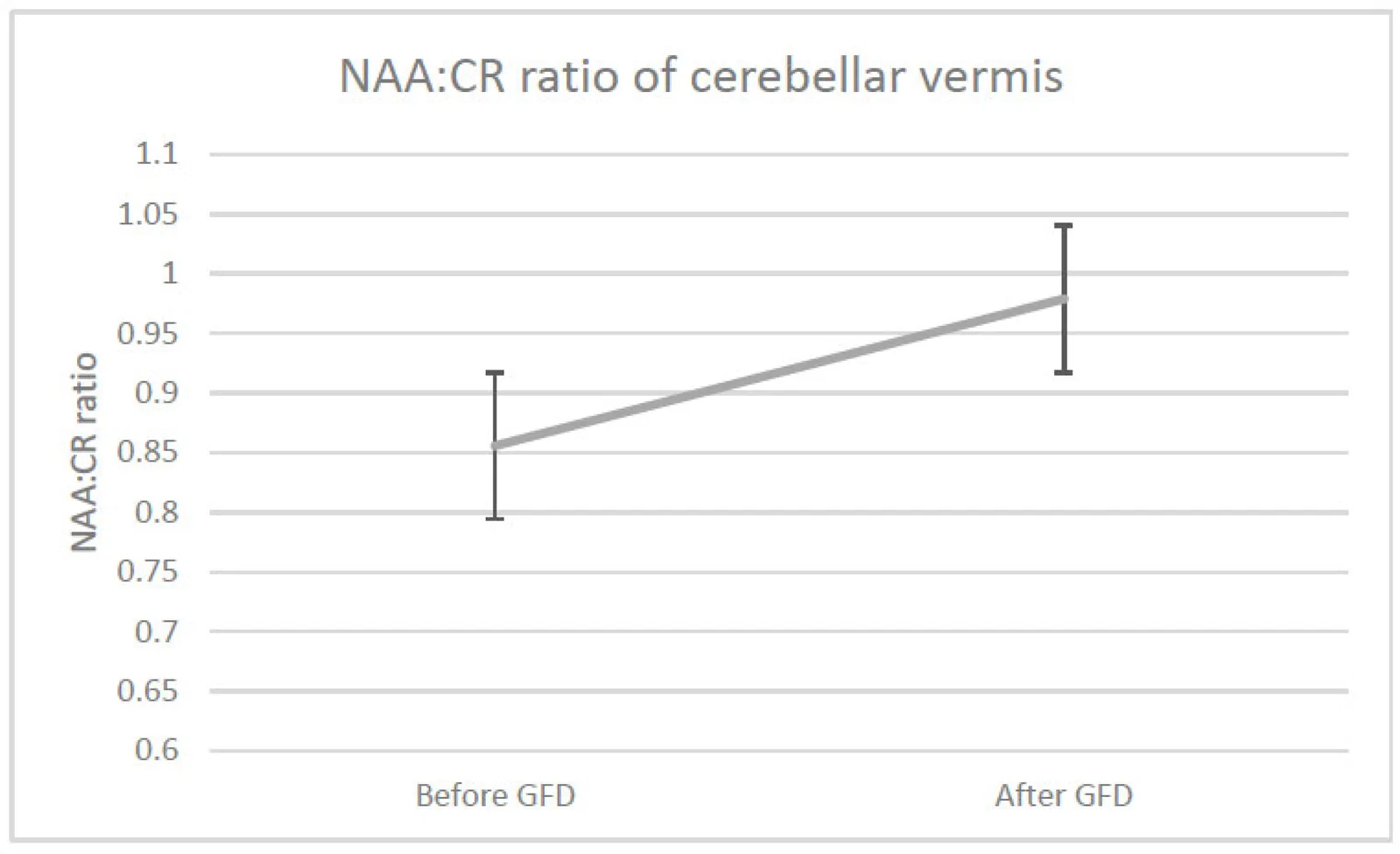
MR spectroscopy of the cerebellar vermis before and after the gluten-free diet in 146 patients with progressive ataxia and positive TG6 antibody in isolation (all other gluten-sensitivity antibodies were negative). *N*-Acetyl Aspartate/Creatine (NAA/Cr) area ratio collectively increased significantly from 0.86 +/− 0.009 to 0.98 +/− 0.009 (*p* < 0.0001 by Student’s *t*-test mean =/− SD). Mean: 22.2 months between each scan, range: 6–80 months.

**Figure 3:**
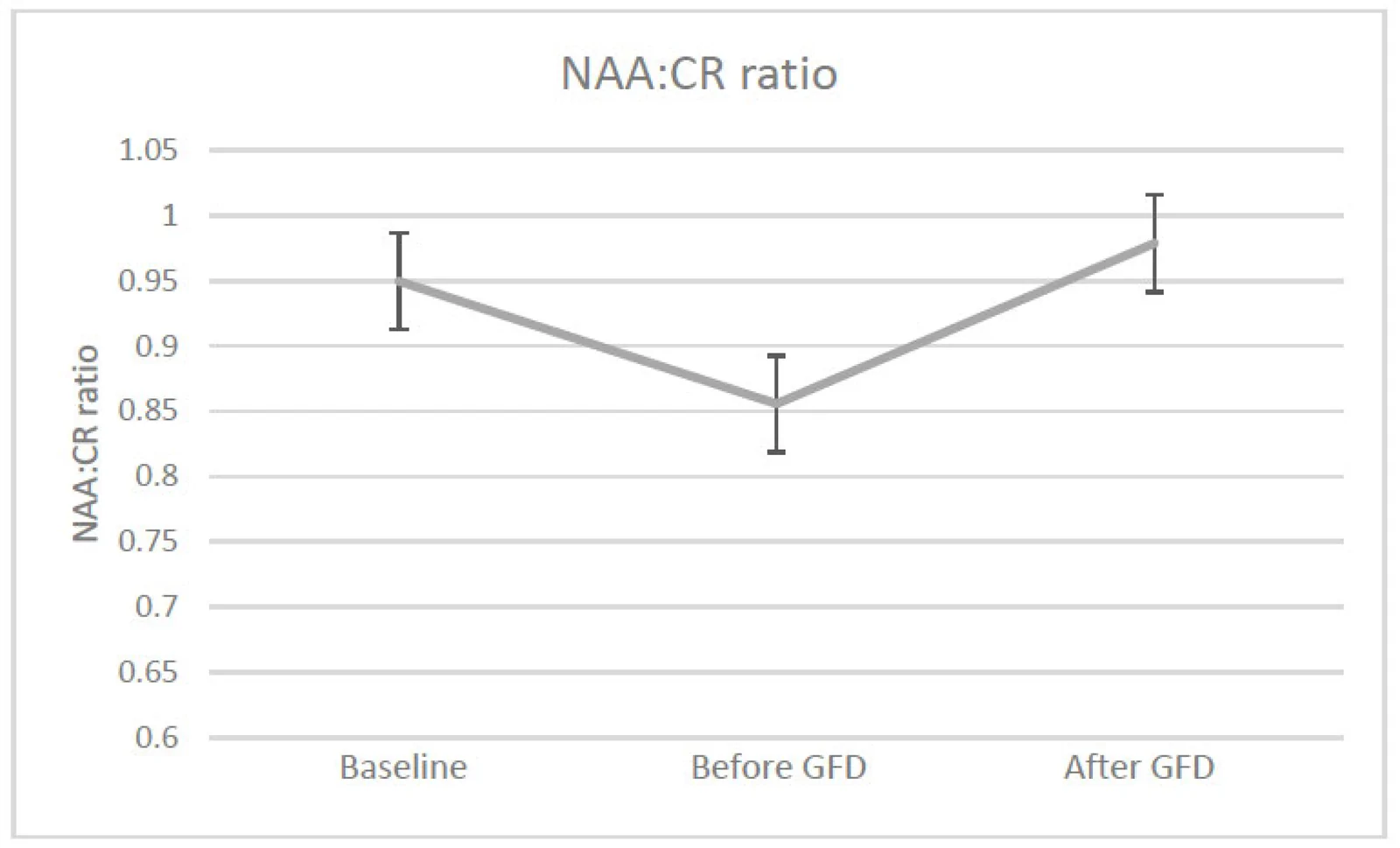
MR spectroscopy of the cerebellar vermis in 47 patients before (baseline/before diet, as they had not yet been diagnosed with GA and continued to consume gluten) and after the diagnosis of GA (with the availability of TG6 testing) and the introduction of a gluten-free diet (before/after diet). The interval between the first 2 scans was 24 months (median), range 4–48, and 18 months between the second 2 scans (median), range 6–36. There is a reduction in the *N*-Acetyl Aspartate to Creatine (NAA/Cr) area ratio over the vermis (baseline to before diet) before the diagnosis of GA (0.95 =/− 0.08, reducing to 0.86 +/− 0.1, mean =/− SD). After TG6 testing became available and the diagnosis of GA was made, there was improvement on GFD (from 0.86 to 0.98 =/− 0.1).

NAA/Cr area ratio from the cerebellar vermis increased in 137/146 (94%) patients (Figure 2); in one patient, the repeat NAA/Cr was the same and in 8 it was worse. In 4 out of these 8 patients, the TG6 antibody titre increased, implying suboptimal adherence to the GFD. Overall, the NAA:Cr ratio increased significantly, from 0.86 +/− 0.009 to 0.98 +/− 0.009 (*p* < 0.0001 by Student’s *t*-test). In 47 out of the 146 patients, more than one MR spectroscopy scan was available prior to the TG6 antibody test availability. All of these patients showed a decline of the NAA/Cr ratio prior to the diagnosis of GA, introduction of GFD (based on TG6 antibody seropositivity) and improvement after GFD (Figure 3). Spearman’s correlation performed on this group demonstrated a very small non-significant positive relationship between change in cerebellar vermis NAA/Cr ratio and duration of the gluten-free diet (r(144) = 0.0755, *p* = 0.365).

### 3.5. Effect of GFD in Patients with Ataxia and Seropositivity for TG6 Antibodies Subsequently Shown to Have a Genetic Cause for Their Ataxia

Over the last 25 years, we have assessed 38 patients with genetic ataxia and seropositive gluten serology (AGA and/or TG6 when it became available). These patients adopted a GFD and underwent subsequent MRS to assess their progress. They were subsequently diagnosed with a genetic ataxia. Although there was a trend of improvement in the MRS of the vermis with the GFD (NAA/Cr vermis 0.83 to 0.87, and NAA/Cr hemisphere 0.87 to 0.89), this was not significant (*p* = 0.0836 for the vermis and *p* = 0.1875 for the hemisphere, paired *t*-test). Amongst this group of 38 patients with genetic ataxia, 16 patients were seropositive for TG6 antibodies alone. The changes in MRS before and after GFD in this group were not significant: NAA/Cr 0.83 to 0.90, *p* = 0.092 for the vermis and 0.89 to 0.93 for the hemisphere, *p* = 0.27 (paired *t*-test). However, there was a significant fall (*p* = 0.016, paired *t*-test) in the serum level of TG6 antibodies (mean 8.738 to 5.515) after the introduction of the GFD.

**Figure 4:**
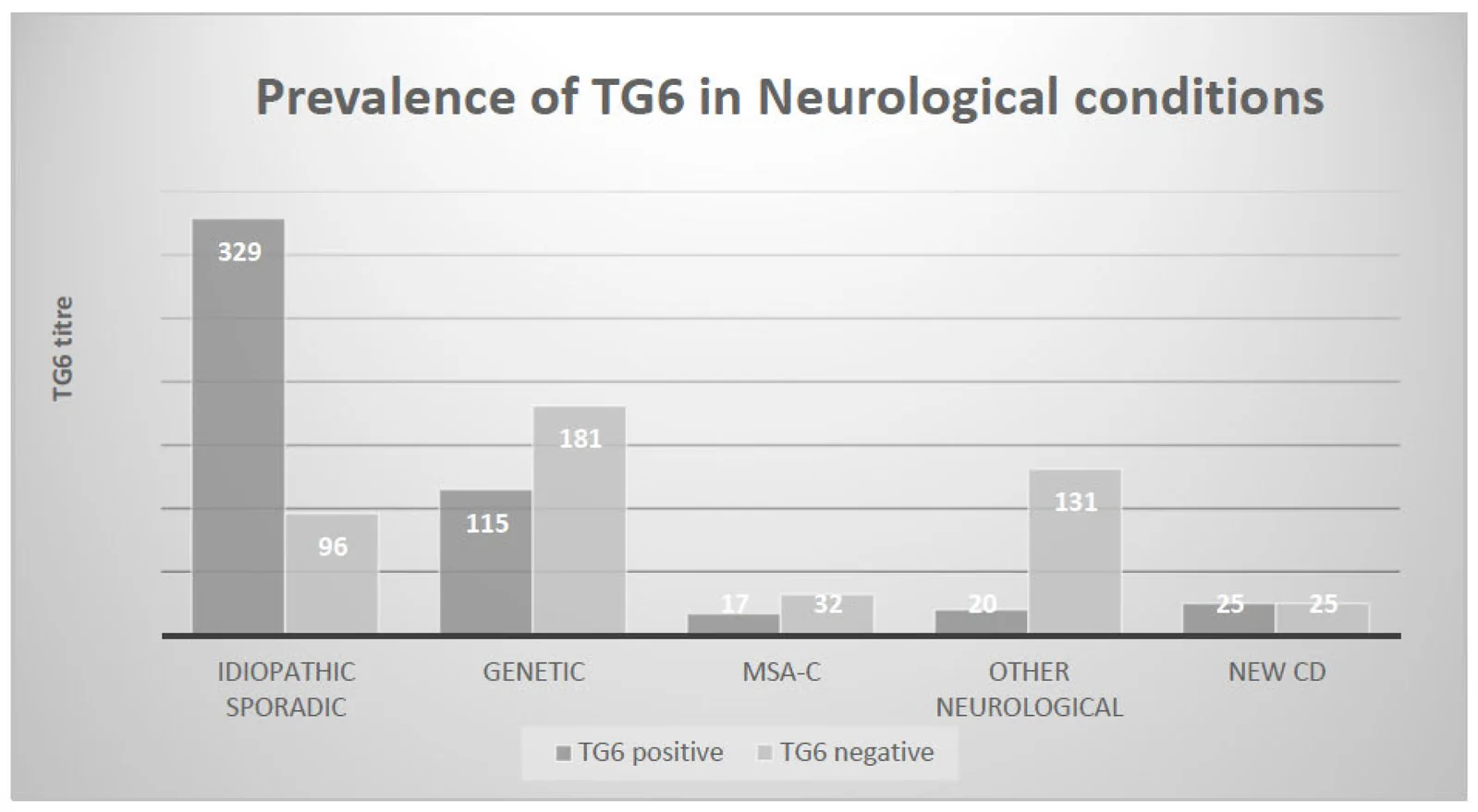
The prevalence of TG6 antibodies in ataxias and other neurological conditions as well as in patients with newly diagnosed coeliac disease (new CD) presenting to the gastroenterologists. Idiopathic sporadic refers to ataxias where no other cause for the ataxia was identified (TG6 positive in 329/425, 77%), genetic refers to genetically confirmed ataxias (TG6 positive in 115/296, 39%), MSA-C refers to patients with cerebellar variant of multiple system atrophy (TG6 positive 17/59, 29%) and other neurological refers to patients with neurological diseases other than ataxia (TG6 positive in 20/151, 13%).

Table 1 summarizes the prevalence of TG6 antibodies amongst the various cohorts and Figure 4 illustrates this prevalence amongst the different populations.

## 4. Discussion

TG6 is a brain-expressed transglutaminase sharing 65% homology with TG2 (TGG) and TG3 [9,10]. All three transglutaminases share the same genetic background and have 65% homology. Patients with GA have been shown to have an immunological response directed to TG6, making them more susceptible to neurological insult. Indeed, previous work from our group has demonstrated that amongst patients with classic CD presenting to the gastroenterologists, 40% were seropositive for TG6. These patients had significantly reduced cerebellar grey matter when compared to those who were negative for TG6 [11]. This suggests that the presence of TG6 is associated with significant brain harm at the time of diagnosis of CD. In this study, using the commercial assay, we found a similar rate of seropositivity to TG6 (50%) amongst patients with newly diagnosed CD presenting with typical gastrointestinal symptoms of CD. It is worth noting, however, that patients presenting with GA may have little or no gastrointestinal symptoms irrespective of the presence of enteropathy.

The introduction of a commercial TG6 assay enabled the immunology laboratory at Sheffield Teaching Hospitals NHS Trust to offer anti-TG6 antibody testing on a routine clinical basis. To our knowledge, our immunology lab is, as yet, the only one to offer anti- TG6 testing in ataxia assessment.

In keeping with our previous observation (using an in-house anti-TG6 assay), we have observed a strong association between AGA and TG6 seropositivity in patients with GA. Ninety-six percent of patients fulfilling the original definition of GA, based on the presence of circulating AGA antibodies in the absence of an alternative cause of progressive ataxia, were seropositive for TG6 antibodies, showing good correlation between AGA and TG6 in the context of GA.

Although there is an unequivocal relationship between gluten sensitivity and cerebellar ataxia, problems remain in case definition. Arguably, a ‘gold standard’ for diagnosis of gluten ataxia is clinical response to a gluten-free diet, but that is unsatisfactory as clinical response may be difficult to measure especially in short-term studies, and patients with long-established ataxia and cerebellar atrophy may not notice any meaningful clinical response. A surrogate measure of response to GFD is cerebellar MR spectroscopy that we have demonstrated to be sensitive.

Using the TG6 assay during routine clinical practice over the last 5 years, we have been able to identify a large number of patients (324 out of 417, 77%) previously labelled as having idiopathic sporadic ataxia who are seropositive for TG6 antibodies without other serological markers for gluten sensitivity. These patients followed a progressive illness course (Figure 2). The recognition that this group had GA only became possible after anti-TG6 antibody testing became available. We have here presented evidence that these patients benefit from the introduction of GFD as indicated by improvement in cerebellar MR spectroscopy. We have also shown here that TG6 antibodies are gluten-dependent, with significant reduction of the titre after the introduction of the GFD. In the context of idiopathic ataxia, TG6 antibody therefore appears to be a more sensitive marker for GA than AGA. It is also worth noting that persistently positive TG6 antibodies (and not necessarily the TG6 titre) in the context of neurology patients who are on GFD (the assumption being that they are not strict with their GFD) is associated with significantly more symptom severity, poorer physical functioning and more regional brain atrophy [12].

The current report, to our knowledge, represents the largest cohort of patients with GA who were seropositive for TG6 yet seronegative for all other gluten-dependent antibodies but successfully responded to GFD. The unequivocal MR spectroscopy response to the GFD and the demonstration that circulating TG6 antibodies are gluten-dependent highlights the utility of this test in the diagnosis and management of GA.

A systematic review and meta-analysis have demonstrated the link between idiopathic sporadic ataxia and the prevalence of AGA, something that our group first reported 30 years ago [2,13]. A few small research studies utilizing TG6 antibodies have been published, but can be criticized for lack of power, poor diagnostic characterization of the ataxia cohorts screened, and statistical shortcomings [8,14]. Such studies also tend to ignore the response of patients with GA to GFD demonstrated in several studies [15,16,17,18,19,20,21,22,23,24].

Our current diagnostic approach is to use all gluten sensitivity-related antibody tests (TG2, EMA, native AGA and TG6) in combination, as each test provides complementary information in relation to the gluten sensitivity spectrum of diseases. TG6 antibodies, however, can be the sole serological marker for GA, making this test an important diagnostic tool.

Of interest is the unexpected finding of high prevalence of TG6 antibodies (39%) in patients subsequently shown to have a genetic cause for their ataxia, something that has been reported once before in a smaller study [25]. This figure was significantly higher than what was found in non-ataxic patients with neurological disorders and in patients with MSA-C, a degenerative, non-genetic form of ataxia. The type of genetic ataxia did not seem to influence the prevalence of TG6 antibodies but the number of patients in each genetic group is too small to exclude a type 2 error.

We were able to show a correlation between the duration of ataxia and TG6 seropositivity amongst patients with SCA6, one of the most common genetic ataxias in the UK. We found a significant difference in the prevalence of TG6 antibodies in those with a long duration (higher prevalence) vs those with a short duration of ataxia (low prevalence). This raises the possibility that anti-TG6 antibodies are generated in response to cerebellar degeneration in the context of a genetic/progressive ataxia. Against this argument is the observation that the TG6 titres reduced following the introduction of GFD, suggesting that these antibodies are, at least in part, gluten-driven. Furthermore, most of these patients were also seropositive for antigliadin antibodies. Another possible explanation may be that cerebellar degeneration in the context of a genetic ataxia may predispose to GS. The duration and severity of the cerebellar insult may also play a role, given that the prevalence of TG6 antibodies in MSA-C, a relatively rapidly progressive ataxia, was significantly less than in genetic ataxias (long duration).

Our results show that in patients with genetic ataxia who are seropositive for TG6 antibodies, adoption of GFD was associated with a trend towards an improvement in the NAA/Cr ratio in the cerebellum. Long-term clinical follow-up suggests that GFD is unlikely to be noticeably beneficial in this group of patients, in contrast to the more substantial and sustained clinical improvement observed in patients with GA. This is possibly because the cerebellar degeneration in patients with genetic ataxia is primarily genetically driven and any potential benefit from GFD is likely to be obscured by the progressive nature of genetic ataxias. Indeed, we propose that lack of improvement with a strict GFD in patients suspected of having GA should raise concern about the possibility of an underlying alternative diagnosis and should therefore help distinguish GA from other patients with ataxia who also happen to be gluten sensitive.

The prevalence of anti-TG6 antibodies in our control group of patients with neurological disorders without ataxia was 13%. We did not collect data on the prevalence of these antibodies (using the same assay) in the healthy population, but from other publications (using the same commercial assay), the prevalence of TG6 IgA in the healthy population was 9% [8]. With the use of our in-house TG6 assay, we found the prevalence of TG6 in the healthy population to be 4%. A small number of published studies have used previous versions of the Zedira TG6 assay and have found the prevalence of TG6 in their healthy control populations to range from 2.7% to 4.3% [26,27,28].

An important question arising from the data presented here is why some patients only have positive serology for TG6 antibodies in the absence of any other gluten-sensitivity-related antibodies. Does this mean that the immunological reaction resulting in neurological damage takes place away from the gut? Against this contention is the fact that the gut is the entry point of gluten, the essential antigen in gluten sensitivity. What is known about the pathophysiology of GA is that patients with GA have TG6 antibodies in the cerebrospinal fluid and that in some of these patients there is also evidence of intrathecal plasma cells (CD138), implying that these antibodies may well be produced intrathecally. This suggests that the immunological response in patients with GA is preferentially concentrated in the CNS, which may explain the absence of other serological markers of gluten sensitivity in the serum. In favour of this theory was also the fact that no correlation was found between serum and cerebrospinal fluid levels of TG6 antibodies.

There are limitations of this study. Firstly, the data presented are retrospective and based on routine clinical practice in a busy national ataxia centre, without any strict eligibility criteria being applied. This might induce some unsuspected bias in case selection. Secondly, it is possible that some of the 77% TG6 seropositive patients labelled as having GA may, in the future, be found to have a novel genetic cause for their ataxia, particularly given that the prevalence of TG6 antibodies is increased amongst patients with genetic ataxias. Against this possibility is the fact that the response to GFD in this group of patients as assessed by MR spectroscopy was significant and sustained.

## 5. Conclusions

Anti-TG6 antibodies appear to be a more sensitive diagnostic marker for GA than native AGA, provided that all other causes of ataxia, including genetic causes, have been excluded. Patients with isolated TG6 seropositivity and progressive ataxia, even if seronegative for all other gluten-sensitivity-related antibodies, may benefit from strict GFD. We suggest that this test is offered to those presenting with progressive ataxia and if seropositive for TG6 and no other cause for ataxia is identified, the patient should be offered a strict gluten-free diet.

## Figures and Tables

**Table 1:** Prevalence of TG6 antibodies in the various cohorts; AGA = antigliadin antibodies, TGG = tissue transglutaminase antibodies, EMA = endomysium antibodies, MSA-C = cerebellar variant of multiple system atrophy, CD = coeliac disease.

Patient Groups and Healthy Cohorts	Number of Patients with Positive TG6 IgA and/or IgG Antibodies/Total Number, (%) IgA TG6 Antibodies/Total Number (%)
Patients with gluten ataxia as defined by positive AGA in the absence of alternative cause for the ataxia, baseline samples	155/161 (96%) IgA and/or IgG TG6 130/161 (81%) IgA TG6
Patients with gluten ataxia already on gluten-free diet (no baseline TG6 available)	236/356 (66%) IgA and/or IgG TG6 191/356 (54%) IgA TG6
Patients with idiopathic sporadic ataxia (all genetic and other causes excluded) negative for AGA, TGG and EMA	329/425 (77%) IgA and/or IgG TG6 272/425 (64%) IgA TG6
Genetically confirmed ataxias	115/296 (39%) IgA and/or IgG TG6 98/296 (33%) IgA TG6
Patients with clinically established MSA-C	17/59 (29%) IgA and/or IgG TG6 11/59 (19%) IgA TG6
Patients with newly diagnosed CD presenting to gastroenterologists	25/50 (50%) IgA and/or IgG TG6 19/50 (38%) IgA TG6
Other neurological disease, no ataxia	20/151 (13%) IgA and/or IgG TG6 17/151 (11%) IgA TG6
Healthy controls	18/194 (9%) IgA TG6 same assay from another study [8]

## Data Availability

Data are included in the article. Further inquiries can be directed to the corresponding author.
